# NARRATIVE MEDICINE AS INTERDISCIPLINARY PRACTICE

**DOI:** 10.1093/geroni/igac059.963

**Published:** 2022-12-20

**Authors:** Ulla Kriebernegg

**Affiliations:** University of Graz, Graz, Steiermark, Austria

## Abstract

When dealing with patients who talk about their illnesses, medical doctors need to interpret the stories they hear. Also, they need to make sense of their own experiences regarding their medical encounters. Narrative and its analysis plays a central role in such processes that require empathy and self-reflection. The interdisciplinary practice of teaching medical students concepts and theories from literary studies as well as discussing literary texts with them can expand their scientific medical understanding. This paper explores the concept of narrative in the context of geriatric medical humanities and narrative medicine, looking at how narrative competence translates into medical practice. Presenting findings from a seminar on Narrative medicine at the Medical University of Graz, Austria, it also addresses some methodological and didactic challenges of this interdisciplinary approach.

